# Not Everything Has a Single Diagnosis: A Case of Hematemesis, Pneumothorax, and Acute Pancreatitis Mimicking Boerhaave Syndrome

**DOI:** 10.7759/cureus.116565

**Published:** 2026-09-19

**Authors:** Neena Cherian

**Affiliations:** 1 Emergency Medicine, Bangalore Baptist Hospital, Bengaluru, IND

**Keywords:** acute pancreatitis, anchoring bias, boerhaave syndrome, cognitive bias, emergency medicine, hickam's dictum, pneumothorax, premature closure, upper gastrointestinal bleeding

## Abstract

Patients presenting with multiple unrelated complaints are a common occurrence in the emergency department and one that poses significant difficulty. While we as physicians are trained to look for a unifying diagnosis, we must never forget “Hickam’s dictum,” stating that a patient may have multiple simultaneous diagnoses.

A 48-year-old man presented to the emergency department with a history of hematemesis, acute shortness of breath, epigastric pain, and right-sided chest pain, after a bout of alcohol binge. On examination, the patient had reduced air entry on the right side, raising suspicion for pneumothorax, which was subsequently confirmed on imaging. The presentation immediately raised suspicion of Boerhaave syndrome given the combination of hematemesis, chest pain, and respiratory distress. However, further clinical assessment and investigation revealed three concurrent pathologies: an upper gastrointestinal bleed, pneumothorax, and acute pancreatitis. The patient was subsequently evaluated and treated for each complaint separately and was discharged from the hospital.

This case highlights the need for us to always be aware of cognitive biases like anchoring bias and premature closure when evaluating patients with complex emergency presentations. Although a unifying diagnosis may provide a seemingly elegant explanation for multiple findings, clinicians should remain alert to the possibility of coexisting diseases.

## Introduction

Emergency medicine frequently requires clinical decisions to be made with incomplete information, often before a definitive diagnosis is available. In this setting, doctors are naturally drawn toward identifying a single diagnosis that can unify the patient's symptoms. This approach is often useful, particularly when dealing with complex presentations, and reflects the clinical application of Occam's razor, the principle that when several findings can be adequately explained by a single diagnosis, the simplest unifying explanation should be considered.

However, the pursuit of diagnostic elegance can itself become a cognitive trap. Not every clinical presentation has a single underlying explanation, and patients may present with multiple concurrent diseases. Hickam's dictum captures this principle beautifully: "A man can have as many diseases as he damn well pleases" [1].

Saint's triad provides a classic illustration of the coexistence of multiple pathological processes without a single underlying mechanism. This principle is particularly relevant to our case, in which the combination of hematemesis, chest pain, and respiratory distress mimicked Boerhaave syndrome. However, further evaluation revealed three concurrent pathologies: right-sided spontaneous pneumothorax, acute pancreatitis, and upper gastrointestinal bleeding. As Hilliard et al. noted, consideration of both Occam's razor and Saint's triad may help physicians provide better care [2].

This principle is particularly relevant in emergency medicine, where diagnostic reasoning is vulnerable to cognitive biases such as anchoring and confirmation bias [3]. While a unifying diagnosis may often be the most efficient explanation, clinicians must remain alert to the possibility that more than one disease process may be responsible for a patient's presentation.

We present a case that illustrates the importance of considering Hickam's dictum and Saint's triad when a seemingly unifying diagnosis does not adequately explain the clinical picture.

## Case presentation

A 48-year-old man with no known comorbidities presented to the emergency department following alcohol intake 20 minutes before presentation. Following alcohol intake, he developed retching, followed by an episode of hematemesis associated with severe right-sided chest pain and epigastric pain. He subsequently developed acute-onset shortness of breath. He had a history of alcohol consumption daily for 20 years. He was a non-smoker, with no previous history of alcohol withdrawal, pancreatitis, gastrointestinal bleeding, or pneumothorax.

In view of his respiratory distress and chest pain, he was triaged as a priority 1 patient. On initial assessment, the airway was patent, although the patient was able to speak only in single words, indicating severe respiratory distress. His respiratory rate was 45 breaths/min, with an oxygen saturation of 88% on room air and markedly increased work of breathing. Examination of the respiratory system revealed reduced air entry on the right side. The trachea was central, and there was no clinically evident subcutaneous emphysema. His heart rate was 129 beats/min and blood pressure was 100/60 mmHg. His Glasgow Coma Scale score was 15/15, and capillary blood glucose was 180 mg/dL. No other significant findings were noted on exposure. Two large-bore intravenous cannulas were secured, fluid resuscitation was initiated, and blood was arranged in view of the reported hematemesis and hemodynamic instability.

Bedside point-of-care ultrasound (POCUS) of the right hemithorax demonstrated an absent lung sliding pattern with a barcode sign and a lung point, suggestive of pneumothorax. A chest radiograph subsequently confirmed a right-sided pneumothorax (Figure 1), following which an intercostal drain was inserted with clinical improvement in respiratory distress.

**Figure 1:**
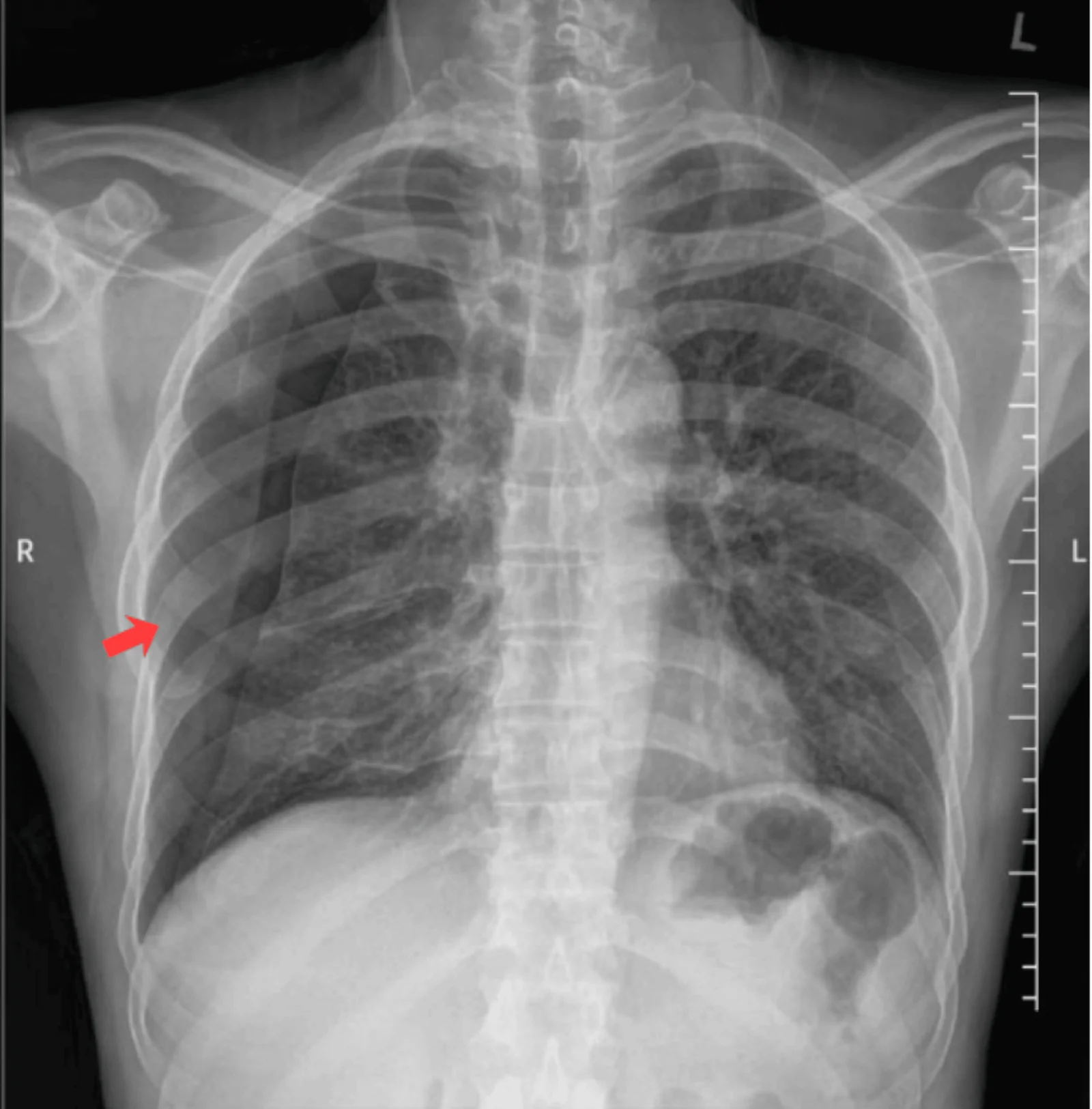
Chest radiograph demonstrating a right-sided pneumothorax with a visible visceral pleural line and peripheral absence of lung markings (red arrow). No significant mediastinal shift is evident.

Following intercostal drain insertion, the patient's respiratory distress improved, and a follow-up chest radiograph demonstrated resolution of the pneumothorax (Figure 2).

**Figure 2:**
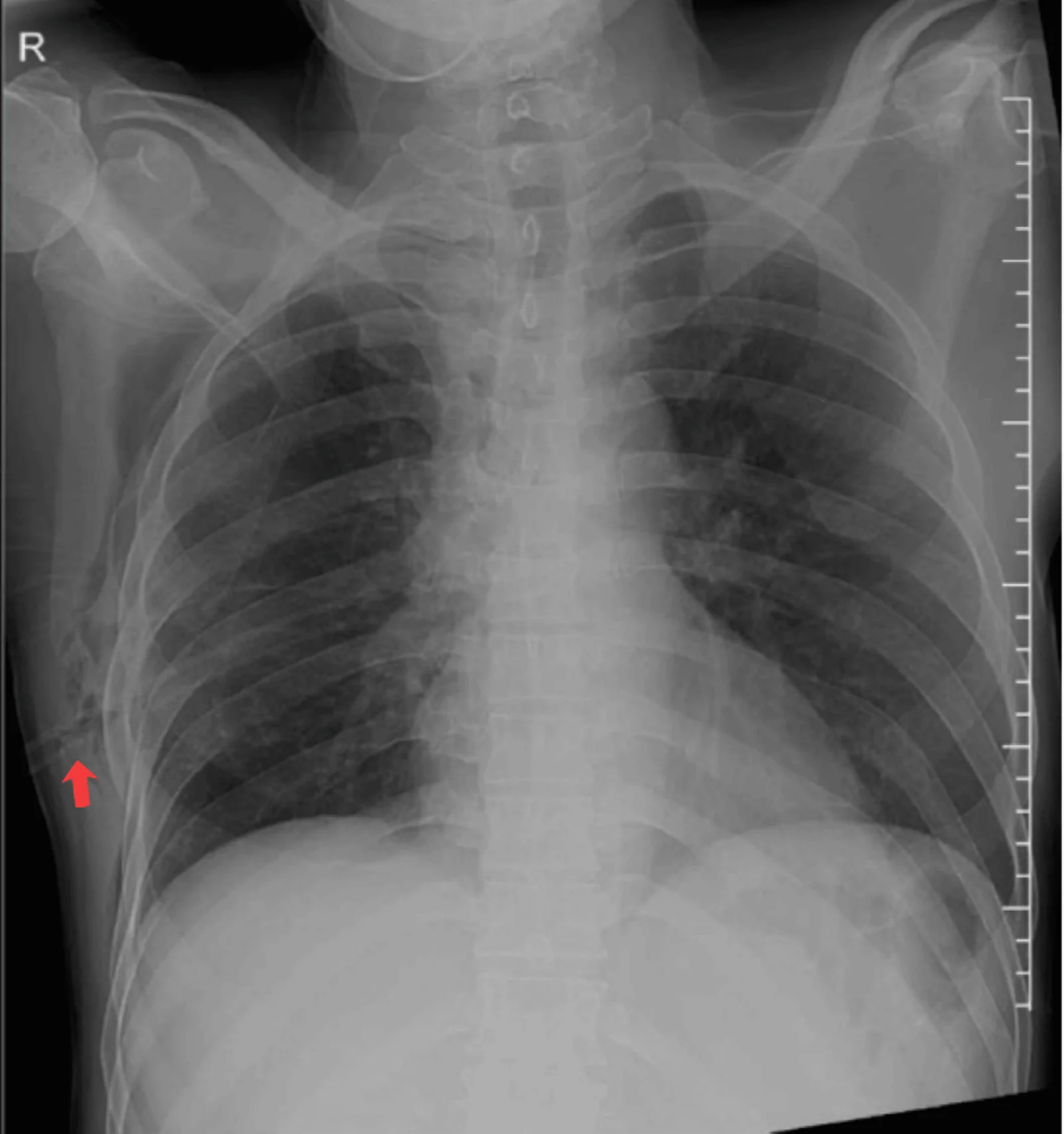
Follow-up chest radiograph after intercostal drain (red arrow) insertion demonstrating resolution of the right-sided pneumothorax. No significant residual pneumothorax is seen.

Given the combination of hematemesis, acute chest pain, and respiratory distress following alcohol intake, esophageal perforation Boerhaave syndrome was considered an important differential diagnosis.

An IV contrast-enhanced CT of the chest and abdomen was therefore performed. The CT demonstrated small right apical bullae and a bulky pancreas with peripancreatic fat stranding (Figure 3). No esophageal perforation, pneumomediastinum, or other radiological features suggestive of Boerhaave syndrome were identified on the CT. Oral contrast was not administered, and CT esophagography or a water-soluble contrast esophagram was not performed, as the patient's subsequent clinical condition did not warrant further dedicated evaluation for esophageal perforation.

**Figure 3:**
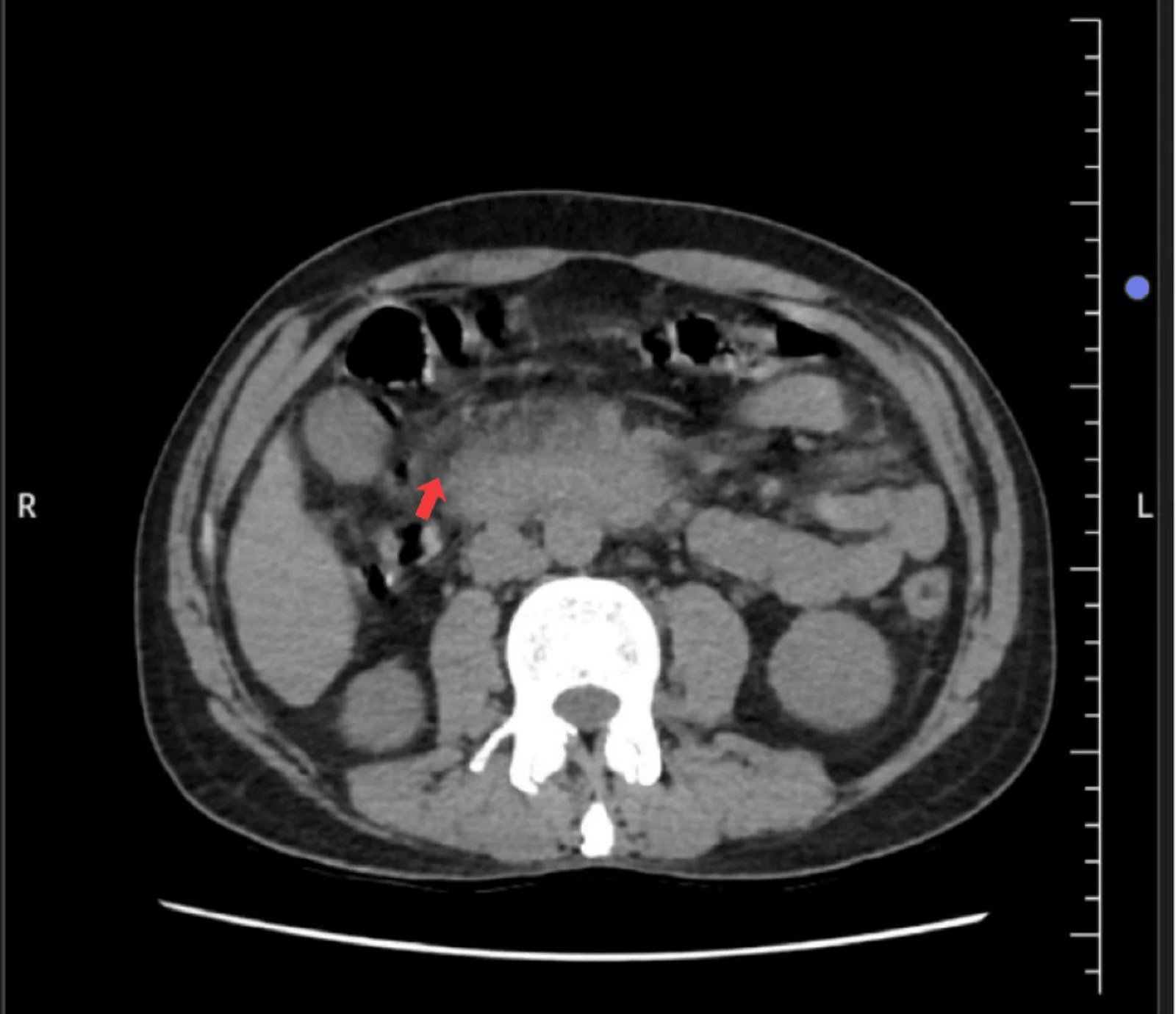
Axial CT image of the abdomen demonstrating a bulky, edematous pancreas with surrounding peripancreatic fat stranding (red arrow), consistent with acute pancreatitis.

The CT findings, together with the characteristic epigastric pain and elevated pancreatic enzymes (Table 1), were consistent with acute pancreatitis. In the absence of another identified precipitant and in the context of recent alcohol intake, the pancreatitis was considered probably alcohol-related, although other etiologies were not comprehensively excluded. The right-sided pneumothorax was considered spontaneous, with small right apical bullae noted on CT.

**Table 1:** Laboratory investigations at presentation. AST: aspartate aminotransferase; ALT: alanine aminotransferase; CRP: C-reactive protein; INR: international normalized ratio

Parameter	Patient value	Reference range
Hemoglobin	14.3 g/dL	13.5-17.5 g/dL
Total leukocyte count	9.0 × 10³/mm³	4.0-10.0 × 10³/mm³
Total bilirubin	2.7 mg/dL	0.3-1.2 mg/dL
Direct bilirubin	0.6 mg/dL	0.00-0.20 mg/dL
Alkaline phosphatase	141 U/L	43-115 U/L
Gamma-glutamyl transferase	520 U/L	0-55 U/L
AST	42 U/L	<50 U/L
ALT	20 U/L	10-40 U/L
Albumin	4.3 g/dL	3.5-5.2 g/dL
Blood urea nitrogen	29 mg/dL	7-25 mg/dL
Creatinine	2.0 mg/dL	0.7-1.4 mg/dL
Sodium	133 mmol/L	136-145 mmol/L
Potassium	3.64 mmol/L	3.5-5.1 mmol/L
Calcium	7.8 mg/dL	8.6-10.3 mg/dL
Lipase	601 U/L	0-67 U/L
Amylase	194 U/L	22-80 U/L
CRP	2.3 mg/L	0-5 mg/L
INR	0.85	0.8-1.2

Upper gastrointestinal endoscopy performed 24 hours after presentation demonstrated pan-erosive gastritis.

Serial hemoglobin measurements demonstrated a decline from 14.3 g/dL at presentation to 11.4 g/dL on day 3, followed by an increase to 13.4 g/dL by day 5 (Table 2). The initial decline was considered likely to be related, at least in part, to fluid resuscitation and hemodilution following the reported gastrointestinal bleeding, rather than evidence of ongoing significant blood loss. The patient did not require blood transfusion during the hospitalization.

**Table 2:** Serial hemoglobin measurements during hospitalization.

Day	Hemoglobin (g/dL)
Day 1 (presentation)	14.3
Day 2	13.4
Day 3	11.4
Day 4	13.2
Day 5	13.4

During the subsequent ICU stay, the patient developed features of alcohol withdrawal syndrome, which was managed with appropriate supportive and pharmacological therapy. His withdrawal symptoms subsequently improved.

Thus, the patient's presentation was ultimately attributed to three concurrent pathologies: upper gastrointestinal bleeding with pan-erosive gastritis, a right-sided spontaneous pneumothorax, and acute pancreatitis, probably alcohol induced.

## Discussion

This case illustrates the limitations of relying on a single unifying diagnosis when evaluating a complex emergency presentation. The combination of hematemesis, acute chest pain, and severe respiratory distress following alcohol intake appropriately raised immediate concern for Boerhaave syndrome. However, subsequent evaluation demonstrated three concurrent pathological processes: right-sided spontaneous pneumothorax, acute pancreatitis, and upper gastrointestinal bleeding secondary to pan-erosive gastritis, which together accounted for the patient's presentation.

The attraction of a unifying diagnosis is understandable in emergency medicine, where clinicians frequently rely on pattern recognition to rapidly identify potentially life-threatening conditions. However, the presence of a memorable clinical syndrome can disproportionately influence diagnostic reasoning. Named syndromes provide useful cognitive frameworks, but their familiarity may cause clinicians to gravitate toward a diagnosis before all available information has been integrated. The rarity of Boerhaave syndrome further illustrates this tension. In a 10-year retrospective review from Middlemore Hospital, only eight cases of spontaneous esophageal perforation were identified [4].

The initial consideration of Boerhaave syndrome in our patient was nevertheless clinically appropriate. Boerhaave syndrome refers to spontaneous transmural rupture of the esophagus, classically occurring following forceful vomiting [5]. In a review of 18 cases, pain was reported in 83% of patients, vomiting in 79%, and dyspnea in 39% [6]. Thus, the combination of hematemesis, chest pain, and severe dyspnea in our patient created a clinically compelling pattern that warranted urgent evaluation for Boerhaave syndrome. Although rare, the condition is associated with substantial mortality, making rapid recognition and diagnosis essential. A recent series of 33 surgically treated patients reported an in-hospital mortality of 24%, emphasizing the potentially lethal nature of delayed or missed diagnosis [7].

However, this same pattern illustrates the potential for anchoring and confirmation bias in emergency medicine. Cognitive biases such as anchoring and confirmation bias are well described among emergency physicians and may contribute to diagnostic error [3]. Diagnostic errors have been reported in approximately 10-15% of emergency department encounters and have been implicated in a substantial proportion of adverse events in hospitals [8]. In our patient, the initial suspicion of Boerhaave syndrome appropriately prompted further investigation; however, the absence of esophageal perforation or pneumomediastinum on CT highlighted the importance of considering alternative diagnoses when a suspected unifying diagnosis does not account for all clinical findings.

This distinction is central to the concepts of Occam's razor and Hickam's dictum. Occam's razor encourages clinicians to seek a single diagnosis capable of explaining the patient's findings, whereas Hickam's dictum reminds us that a patient may have multiple diseases simultaneously [1]. Saint's triad similarly demonstrates that unrelated pathological processes may coexist without requiring a common underlying mechanism [2]. The clinical challenge is therefore not to abandon parsimony, but to recognize when the pursuit of parsimony has become an assumption rather than a conclusion.

The rarity of genuinely independent concurrent diagnoses is highlighted by the analysis of Hickam's dictum by Aberegg et al. Among 83 published case reports described as illustrating Hickam's dictum or violating Occam's razor, most additional diagnoses represented incidental findings, pre-existing disease, or diseases causally related to the primary diagnosis. Only three cases (3.6%) involved a causally unrelated second disease that contributed to the acute presentation. Furthermore, when clinicians evaluated hypothetical cases involving multiple diagnoses, 79% considered the coexistence of a causally unrelated second diagnosis to be the most unlikely or surprising diagnostic combination [9]. Our case represents this particularly challenging situation, in which multiple independent pathological processes contributed to a single acute presentation.

The case also demonstrates the importance of maintaining a structured diagnostic approach despite an initially compelling diagnosis. Each investigation therefore addressed a different component of the presentation rather than simply searching for confirmation of a single diagnosis.

The principal lesson from this case is not that clinicians should routinely seek multiple diagnoses. Occam's razor remains an important principle in clinical reasoning and is particularly valuable when a single diagnosis adequately explains the clinical findings. However, when important findings remain unexplained, clinicians should resist premature closure and reconsider whether more than one pathological process may be present. As Miller aptly observed, "In my experience, Hickam often triumphs" [1]. In emergency medicine, recognizing when Hickam's dictum has overtaken Occam's razor may be critical to avoiding diagnostic error.

## Conclusions

Complex emergency presentations may not always be explained by a single unifying diagnosis. In this case, the simultaneous presence of a right-sided spontaneous pneumothorax, acute pancreatitis, and upper gastrointestinal bleeding with pan-erosive gastritis initially mimicked the potentially life-threatening diagnosis of Boerhaave syndrome. While rapid recognition of life-threatening conditions remains essential, clinicians should remain alert to findings that are not adequately explained by the initial diagnostic framework. Hickam’s dictum serves as an important reminder that multiple concurrent diseases may coexist, and maintaining diagnostic flexibility may help avoid premature closure and facilitate recognition of additional or alternative diagnoses.
